# Silica Nanoparticles Decrease Glutamate Uptake in Blood–Brain Barrier Components

**DOI:** 10.1007/s12640-024-00696-1

**Published:** 2024-03-04

**Authors:** Fredy Sánchez-Cano, Luisa C. Hernández-Kelly, Arturo Ortega

**Affiliations:** Laboratorio de Neurotoxicología, Departamento de Toxicología, Centro de Investigación y de Estudios Avanzados del Instituto Politécnico Nacional, Av. IPN 2508, San Pedro Zacatenco, 07300 CDMX México

**Keywords:** Blood–brain barrier, Glutamate, Endothelial cells, Astrocyte

## Abstract

Glutamate is the major excitatory amino acid in the vertebrate brain, playing an important role in most brain functions. It exerts its activity through plasma membrane receptors and transporters, expressed both in neurons and glia cells. Overstimulation of neuronal glutamate receptors is linked to cell death in a process known as excitotoxicity, that is prevented by the efficient removal of the neurotransmitter through glutamate transporters enriched in the glia plasma membrane and in the components of the blood–brain barrier (BBB). Silica nanoparticles (SiO_2_-NPs) have been widely used in biomedical applications and directed to enter the circulatory system; however, little is known about the potential adverse effects of SiO_2_-NPs exposure on the BBB transport systems that support the critical isolation function between the central nervous system (CNS) and the peripheral circulation. In this contribution, we investigated the plausible SiO_2_-NPs-mediated disruption of the glutamate transport system expressed by BBB cell components. First, we evaluated the cytotoxic effect of SiO_2_-NPs on human brain endothelial (HBEC) and Uppsala 87 Malignant glioma (U-87MG) cell lines. Transport kinetics were evaluated, and the exposure effect of SiO_2_-NPs on glutamate transport activity was determined in both cell lines. Exposure of the cells to different SiO_2_-NP concentrations (0.4, 4.8, 10, and 20 µg/ml) and time periods (3 and 6 h) did not affect cell viability. We found that the radio-labeled D-aspartate ([^3^H]-D-Asp) uptake is mostly sodium-dependent, and downregulated by its own substrate (glutamate). Furthermore, SiO_2_-NPs exposure on endothelial and astrocytes decreases [^3^H]-D-Asp uptake in a dose-dependent manner. Interestingly, a decrease in the transporter catalytic efficiency, probably linked to a diminution in the affinity of the transporter, was detected upon SiO_2_-NPs. These results favor the notion that exposure to SiO_2_-NPs could disrupt BBB function and by these means shed some light into our understanding of the deleterious effects of air pollution on the CNS.

## Introduction 

Glutamate is one of the most abundant amino acids in the encephalon, and it has been involved in a wide variety of functions. In addition to its role as a protein building block, it is the principal mediator of sensory information, motor coordination, emotions, and cognition, including learning and memory (Petroff 2002). It is needed for the synthesis of key molecules, such as glutathione and polyglutamated folate cofactors. Within the CNS, glutamate is the major excitatory neurotransmitter (Brosnan and Brosnan 2013) and plays critical roles in physiological functions such as synaptic plasticity, neurogenesis, and neurodegeneration. This neurotransmitter is produced through two pathways, which result in the overall conversion of 2-oxoglutarate, to glutamate. One route is the reductive amination of 2-oxoglutarate with ammonium via glutamate dehydrogenase. The second route is through glutamate synthase which catalyzes reductive amination of 2-oxoglutarate using glutamine as the nitrogen donor. Glutamate is metabolized by the action of glutamine synthetase, in the glial cells, continuously reconverted to α-ketoglutarate and metabolized through the tricarboxylic acid cycle. Indeed, is a precursor for γ-aminobutyric acid (Walker and Donk 2016). Glutamate is stored in vesicles in the synapses of glutamatergic neurons and released into the synaptic clef upon nerve stimulation. Prolonged elevated concentrations of glutamate are cytotoxic since overstimulated glutamate receptors, followed by activation of enzymatic cascades and, eventually, cell death, a phenomenon known as excitotoxicity (Iovino et al. 2020). To maintain a proper synaptic transmission, extracellular glutamate concentrations must be kept in the low µM range (Chen et al. 2015). Despite that almost all the cells in the CNS participate in extracellular glutamate removal; astrocytes are, by far, the most efficient cell type in this process, removing around 90% of the glutamate released after an action potential. Glutamate uptake is achieved through two main classes of glutamate transporters, Na^+^-independent and Na^+^-dependent transporters (Mahmoud et al. 2019). Glutamate extra-synaptic levels are regulated mainly by the family of Na^+^-dependent glutamate transporters, known as excitatory amino acid transporters (EAATs) (Danbolt 2001). EAATs expression is highly regulated at several levels from transcription to posttranslational modifications and trafficking to the plasma membrane (Rodríguez-Campuzano and Ortega 2021). Five subtypes of transporters have been described and named EAATs 1 to 5. EAAT1 and EAAT2 are glia-specific, while EAAT3, 4, and 5 are present in neurons. However, at the BBB level, EAAT1, 2, and 3 have been described to move out of the brain glutamate and aspartate (O'Kane et al. 1999; Zlokovic 2008).

Recent studies have indicated that the brain endothelium of the BBB might also play an active role in the regulation of brain glutamate concentrations. Brain endothelial cells have been proposed to act as the efflux route for glutamate through the concerted actions of EAATs, expressed on abluminal (brain-facing) and luminal (blood-facing) membranes (Cohen-Kashi-Malina et al. 2012; Zlotnik et al. 2012). The uptake of glutamate is thermodynamically unfavorable; therefore, 3 Na^+^ ions and 1 proton (H^+^) are needed to be co-transported together with each glutamate molecule, and the efflux of one potassium (K^+^) is compulsory to drive the uptake (Ryan et al. xxxx). These transporters prevent the over-activation of glutamate receptors, recycle the neurotransmitter, and by these means protect the brain from excitotoxicity, a phenomenon that is the biochemical signature of most neurological disorders, which in most of the cases are also related to environmental factors (Dosunmu et al. 2007; Madhaw and Kumar 2023). Recent studies have reported that exposure to nanosized particles is associated with the development of behavioral deficits such as learning and memory (Ranft et al. 2009; Wang et al. 2017).

Natural silica and silicates are crystalline components abundant in the Earth’s crust. Synthetic silica is amorphous and produced in great quantities for commercial purposes and recently for medical applications, making man-made silica nanoparticles the main nanoparticles on Earth (Croissant et al. 2020). In ambient air, circulating air in households and workplaces, these nanomaterials have been found (Brouwer 2010), as well as in airborne pollutants (mineral dust and particulate matter) (Geysen et al. 2004). Due to their unique characteristics such as large surface area, high structural stability, easy surface functionalization, low cost of production, and excellent biocompatibility, SiO_2_-NPs have gained attention in various areas, including biomedical field, imaging, cell tracking, and photothermal therapy (Tang and Cheng 2013). However, their unique characteristics allow SiO_2_-NPs to cross several biological barriers and easy access to the whole body, and therefore each organ (Barua and Mitragotri 2014). Such permeability is evidenced by silica and titanium dioxide nanoparticles that cross the placenta, liver, and brain barrier (Yamashita et al. 2011; Kim et al. 2006). The cellular mechanism of SiO_2_-NPs’ interaction with tissues and barriers is still incomplete. This is particularly relevant for the CNS, a field in which nano neuromedicine is a good candidate for novel applications. The brain is a vulnerable organ due to its limited regenerative capacity; thus, it is protected by an interface that helps it to avoid exogenous insults, the BBB. Once SiO_2_-NPs reach the CNS, interact with neuronal cells (Gilardino et al. 2015), and give rise to neurotoxic effects. Therefore, a better understanding of the biological interaction of SiO_2_-NPs on BBB is of great importance.

The BBB has selective access to the brain and plays a critical role in the supply of the necessary nutrients for a proper neuronal environment and brain homeostasis. In physiological conditions, the structure of BBB is formed by brain microvessel endothelial cells (BMECs), pericytes, and astrocytes, supported by microglia and neurons (Sanchez-Cano et al. 2021). In vitro, multiple variants of the BBB model exist, but these rely on the same principle. The capacity of a molecule to cross a cell monolayer seeded on an insert-transwell system is assessed. Frequently, microvascular endothelial cells are used (HBEC) to form one compartment that mimics the blood and one compartment that mimics the brain site (Santa-Maria et al. 2022). Co-culture models are frequently employed to improve in vitro cellular systems due to their ability to enhance physiological functions and permeability. Astrocytes, which interact and cover almost all the cerebrovasculature, induce and maintain brain endothelial characteristics (Cader 2022; Abbott et al. 2006). The first studies about how glutamate extracellular levels could be regulated by BBB components were made in endothelial cells from bovine tissue and astrocytes from rats (Helms et al. 2012), so the use of cells gives insight into how nanosized particles damage the BBB and its function. In terms of glutamate levels, brain endothelial cells and astrocytes have a strategic location, since these cells express EAATs in the BBB to help keep in low concentrations this neurotransmitter. The idea that the CNS might also be targeted by nanosized particles including air pollutants was first proposed by Oberdorster and Utell (2002), who demonstrated that particulate matter (PM) can cross the blood-air barrier of the lungs, access to the circulatory system and thus, involved in the adverse cardiovascular effects (Oberdörster and Utell 2002). Nanosized material can cross the BBB and enter human and animal brains (Mohan Kumar et al. 2008; Block and Calderón-Garcidueñas 2009).

Using in vitro BBB models, it has been shown that SiO_2_-NPs can cross and alter the permeability in a particle size-dependent manner, and transcellular transport was thought to be the mechanism for the SiO_2_-NPs crossing (Ye et al. 2015). In another study, it was  reported that SiO_2_-NPs could induce tight junction loss and cytoskeleton arrangement and increase the inflammatory response, like that of the vascular endothelial growth (VEGF) factor of BMECs, which actives astrocytes to amplify the generation of VEGF triggering aquaporin-4 expression, thus, causing BBB disruption via an immunoregulatory loop between BMECs and astrocytes after SiO_2_-NPs exposure (Liu et al. 2017). In vivo, reports provide controversial results. Cerebral perfusion or dermal administration of SiO_2_-NPs cross the BBB and reach CNS tissue (Jampilek et al. 2015). In sharp contrast, the dermal and oral exposure to SiO_2_-NPs did not enter the rat brain (Shim et al. 2014). Moreover, few studies have focused on possible SiO_2_-NPs’ interaction and toxic effects on BBB, and therefore, their relationship with brain pathology is scarce. In ischemia, a disease in which there is a glutamatergic disruption, Liu et al. (2015) demonstrated that nanosized particles (PM2.5) disrupt BBB integrity and get access to the CNS. Once in the brain, increased extracellular levels of glutamate are evident after the PM2.5 exposure. Furthermore, pre-treatment with NDMA receptor antagonist MK801 dimishes neuronal loss, suggesting that PM2.5 neurotoxicity is mediated by glutamate (Liu et al. 2015).

Nowadays, the research is focused on the SiO2-NPs’ ability to penetrate the BBB for the transport of therapeutic agents. Indeed, the study of these types of nanoparticles could help to understand the effects of exposure to high levels of particular matter present in the pollution. However, few efforts have focused on the effects of SiO_2_-NPs in the disruption of BBB transport systems. In the present study, we decided to investigate whether SiO_2_-NPs exposure disrupts the glutamate transport system expressed by BBB cell components. First, we focused on the cytotoxic effect of SiO_2_-NPs in HBEC and U-87MG cell lines. Next, we characterized the glutamate transport system expressed in both cell lines (based on activity assays) by using EAAT substrates (Glutamate and Aspartate) and EAAT inhibitors (TBOA and dyhidrokainic acid). Then, kinetics parameters were determined, and the exposure effect of SiO_2_-NPs on glutamate transport activity was evaluated in both cell lines. Exposure of the cell lines to different SiO_2_-NP concentrations (0.4, 4.8, 10, and 20 µg/ml) and time periods (3 and 6 h) resulted in no changes in cell viability. We found that the [^3^H]-D-Asp uptake is sodium-dependent, at least in part, and downregulated by its own substrate (glutamate). Furthermore, SiO_2_-NPs exposure on endothelial and astrocytes decreases [^3^H]-D-Asp uptake at different concentrations (2.4, 4.8, 6.4, and 10 µg/ml). Interestingly, a decrease in the transporter catalytic efficiency, probably linked to a decrease in the affinity of the transporter, was detected upon SiO_2_-NPs exposure. These results demonstrate that SiO_2_-NPs disrupt BBB function and shed light on our current understanding of the deleterious effects of air pollution on the CNS.

## Materials and Methods

### Materials

SiO_2_-NPs ranging from 10 to 20 nm in size, dimethyl sulfoxide (DMSO) (#M81802), and 3‐(4,5‐dimethyl‐2‐thiazolyl)‐2,5‐diphenyl-2H-tetrazolium bromide (MTT; # M2128) were obtained from Sigma–Aldrich (St. Louis, MO, USA). [^3^H]-D-aspartate was purchased from PerkinElmer (Boston, MA). Cell culture medium was from Thermo Fisher Scientific (Carlsbad, CA), and plasticware was purchased from Corning (New York, NY).

### Cell Culture and Silica Nanoparticles Stimulation Protocol

Endothelial cells form the primary structure of the BBB since these cells control the passage of molecules inside and outside the brain (Alahmari 2021). The barrier function of the endothelial cells is mainly provided by tight junctions (TJs) and several transport systems (Sweeney et al. 2018). As it was mentioned before, the BBB is a complex set of cells in which astrocytes participate actively, for example, astrocytes clear neurotransmitters (Danbolt 2001), produce glutathione (Dringen et al. 2015), synthesize and release trophic factors (Nuriya and Hirase 2016), and contribute to neurovascular coupling by extending end-feet processes to the vasculature (Abbott et al. 2006). Results from co-culturing experiments demonstrate that brain endothelial cell contact with astrocytes is required for GLT-1 and GLAST transporter expression (Lee et al. 2017).

Thus, several human cell lines have been used in BBB studies (Eigenmann et al. 2013; Weksler et al. 2013), but the most characterized are human cerebral microvascular endothelial cells (hCMEC/D3) (Weksler et al. 2013) and human brain microvascular endothelial cells (hBMEC) (Eigenmann et al. 2016). However, these models grow with a cocktail of adjuvants, and it is known that adjuvants have an impact on transporter expression (Eisenblätter and Galla 2002; Wedel-Parlow et al. 2009). So, in this work, we chose a human brain endothelial cell line (HBEC-5i) that can grow in a monolayer and mimic the BBB (Puech et al. 2018). Furthermore, this cell line has been cultivated with human astrocytes due to an improvement of the barrier properties (Abbott et al. 2006) by the close interactions between brain endothelial cells and astrocytes (Abbott et al. 2006; Helms et al. 2016); thus, these cell lines could represent the BBB and how the SiO_2_-NPs might disrupt it.

HBEC-5i endothelial cells and U-87MG astrocyte cells were obtained from ATCC; no. CRL-3245 and HTB-14, respectively, Manassas, VA, USA. Initially, HBEC-5i were cultured in Dulbecco’s Modified Eagle Medium, (DMEM-F12 HAM, no. 12400–016, Gibco), supplemented with 10% fetal bovine serum (FBS), 40 µg/ml microvascular growth supplement (MVGS; no. S00525, Gibco), and 1% of antibiotic solution, and U-87MG cells were cultured in Dulbecco’s Modified Eagle Medium, (DMEM-F12 HAM), supplemented with 10% fetal bovine serum FBS and 1% of antibiotic solution. Both cell lines were cultured, seeded, and stimulated separately. The HBEC-5i cell line was cultured on dishes coated with 0.1% gelatin (no. G2500, Sigma–Aldrich), incubated at 37 °C for ≥ 40 min, and then gelatin was aspirated before adding cells to the dishes. For the transport assays, to avoid the cells being detached, the HBEC-5i cell line was also seeded on dishes coated with 0.1% gelatin. Confluent monolayers of both cells (HBEC-5i and U-87MG) were treated with SiO_2_-NPs diluted in DMEM-F12 containing 0.5% FBS, at different concentrations and periods detailed below, based on the data of invitro experiments about SiO_2_-NPs neurotoxic effect (Orlando et al. 2017; Wang et al. 2011). The dilutions of SiO_2_-NPs were previously sonicated before treating the cells, by using a bath sonicator at room temperature for 15 min at 40 W to avoid SiO_2_-NPs agglomeration, as it was described previously (Rodríguez-Campuzano et al. 2020).

### Methods

Cell viability was evaluated by the 3-(4,5-dimethylthiazol-2-yl)-2,5-diphenyltetrazolium bromide (MTT) assay (MTT; # M2128), which determines the ability of metabolically active cells to produce a purple formazan salt after the cleavage of the tetrazolium ring of a yellow substrate (MTT) by mitochondrial reduction (Denizot and Lang 1986). The amount of formazan was determined at *λ* = 560 nm and it is directly proportional to the number of viable cells. Briefly, HBEC-5i and U-87MG cells were seeded in 96-well plates (1 × 10^5^ cells/well) and cultured to an 80 to 90% confluence; cells were treated with vehicle (control), different SiO_2_-NP concentrations (0.4, 4.8, 10, and 20 µg/ml), and periods (3 and 6 h) at 37 °C. Then, 3 h before the SiO_2_-NPs treatment ended, 20 µL/well of an MTT stock solution (0.5 mg/ml) was added directly into each well, and the plates were returned to the incubator. Finally, the medium was discarded, and 180 µL of DMSO was added to each well to dissolve the formazan crystals. Absorbance was measured with a microplate reader (Epoch, BioTek Instruments, VT, USA). Cell viability was calculated as follows: cell viability (%) = average OD of treated wells/average OD of control wells. Three independent experiments (*n* = 3) were performed in quadruplicate from three different passages.

### Neutral Red Uptake Assay

This assay was performed as described previously (Repetto et al. 2008). This test is based on the use of a cationic probe (neutral red) which is taken up into cells by membrane diffusion where it becomes an ion trapped within the lysosomal compartment. Briefly, both cell lines were plated in a 96-well culture plates (1 × 10^5^ cells/well) and treated with vehicle (control); different SiO_2_-NP concentrations ranging from 0.4, 4.8, 10, and 20 µg/ml, for 3 and 6 h; then, the medium of stimulation was discarded; and the cells were washed with 150 µl PBS per well. One hundred microliter of the neutral red medium was added to each well. The plates were incubated for 2 h at the appropriate culture conditions (37 °C). After that, the neutral red medium was removed; the cells were washed with 150 µl PBS, per well; and the washing solution was removed by gently tapping. Neutral red destain solution (50% ethanol 96%, 49% deionized water, 1% glacial acetic acid) was added (150 µl) per well, and the plate was shaken rapidly on a microtiter plate shaker for 10 min until obtaining a homogenous solution. The absorbance of dye was measured using a microplate reader at a wavelength of 570 nm. Three independent experiments (*n* = 3) were performed in quadruplicate from three different passages.

### [^3^H]-D-Aspartate Uptake

The uptake of [^3^H]-D-aspartate (used as a non-metabolizable analogue of L-glutamate) was performed as previously described (Ruiz and Ortega 1995). Cells were seeded in 24 (5 × 10^5^ cells/well) or 48 well plates (2.5 × 10^5^ cells/well). Briefly, the medium was replaced with a pre-warmed uptake buffer containing 25 mM HEPES, 130 mM NaCl, 5.4 mM KCl, 1.8 mM CaCl_2_, 0.8 mM MgCl_2_, 33.3 mM glucose and 1 mM NaH_2_PO_4_, pH 7.4, and 0.4 μCi/mL [^3^H]-D-aspartate ([^3^H]-D-Asp) (specific activity: 12.2 Ci/mmol, Perkin Elmer, MA, USA) (50 μM final aspartate concentration). Uptake was finished by the addition of ice-cold uptake buffer, and cells were lysed with 0.1 N NaOH for 2 h at room temperature. Aliquots of 10 μL were used for protein quantification, and the samples were transferred to scintillation vials, a liquid scintillation cocktail, and 50 μL of glacial acetic acid (to quench chemiluminescence) was added, and radioactivity was measured in a scintillation counter (PerkinElmer, MA, USA). Radioactivity counts were adjusted for protein quantity and calculated as [^3^H]-D-aspartate pmol/(mg protein min^−1^). Three independent experiments (*n* = 3) were performed in quadruplicates (4 wells by condition or group) from three different passages.

#### Glutamate Transport System Characterization

In the case of pharmacological characterization of the glutamate transport system, the cells were pre-treated for 30 min with selective excitatory amino acid transporter (EAAT) 2 blocker, dihydro kainic acid, (DHK) 100 μM, and TBOA 100 μM, a non-specific potent inhibitor of EAAT1,2, and 3. Also, we used glutamate (10 μM, 100 μM, 500 μM, and 1 mM) or aspartate (Asp 1 mM), because it has been shown their substrates downregulate the activity of excitatory amino acid transporters. Then we measured the uptake of [^3^H]-D-aspartate as we indicated previously, in the presence or absence of sodium (Na^+^/Na^−^, Figs. 2 and 3) since glutamate transport is electrogenic (Grewer and Rauen 2005). Three independent experiments (*n* = 3) were performed in quadruplicates from three different passages.

#### Kinetic Parameters of the Glutamate Transport System

For the determination of the kinetic constants, *K*_m_ and *V*_max_, both cell lines were treated with uptake buffer containing 0.4 μCi/mL [^3^H]-D-aspartate + different unlabeled D-Asp concentrations 0,10, 25, 50, 100, and 200 μM (Sigma–Aldrich, MO, USA) (Fig. 4a and b) or pre-treated with DHK 100 μM 30 min before replacing the medium with [^3^H]-D-aspartate + different unlabeled D-Asp concentrations (Fig. 4c and d). Uptake was stopped after 30 min of incubation by washing the cells with an ice-cold uptake buffer, and the samples were processed as described above. A robust nonlinear regression was used to fit a model to the experimental data and estimated the parameters of the Michaelis–Menten equation (GraphPad Prism Software, La Jolla California, USA). Three independent experiments (*n* = 3) were performed in quadruplicates (4 wells by condition or group) from three different passages (Figs. 4 and 5).

#### Effect of SiO_2_-NPs on Glutamate Transporter Systems

To evaluate the effect of SiO_2_-NPs on glutamate transporter systems, we used different concentrations of nanoparticles (2.4, 4.8, 6.4, and 10 μg/ml) which have been demonstrated to be the closest physiologically relevant to SNC exposure (Xie et al. 2010; Wu et al. 2011). Also, in our group, Rodríguez–Campuzano et al. showed that exposure to SiO_2_-NPs at these doses affects protein synthesis in glial cells (Rodríguez-Campuzano et al. 2020). Recent studies have reported that exposure to SiO_2_-NPs activates a pro-inflammatory response, oxidative stress, and unfolded protein production (Wang et al. 2011; Wu et al. 2011; Nemmar et al. 2016), which results in cell death in the CNS, leading to an increase in the release of glutamate, over-activating its receptors, and saturating the excitatory amino acid transport system, triggering an ion imbalance that proceeds neuronal lysis, lasting in cell death cascades (Davide et al. 2018). Indeed, in a pilot experiment, we observed a decrease in the [^3^H]-D-aspartate uptake after the exposure of SiO_2_-NPs 4.8 μg/mL (data not shown). So, both cells were treated with a vehicle (control); Asp 1 mM; or different concentrations of SiO_2_-NPs (2.4, 4.8, 6.4, and 10 μg/ml) for 30 min. After the treatment, cultures were incubated with uptake buffer containing 0.4 μCi/mL [^3^H]-D-aspartate + unlabeled D-Asp 50 μM (Sigma–Aldrich, MO, USA). Uptake was stopped after 30 min of incubation by washing the cells with an ice-cold uptake buffer, and the samples were processed as described above. Four independent experiments (*n* = 4) were performed in quadruplicates from four different passages.

Some experiments were performed in the presence or absence of DHK (100 μM), and TBOA (100 μM) was pre-incubated 30 min before being exposed to SiO_2_-NPs (4.8 μg/mL), vehicle, or Asp 1 mM. After the treatment, the cultures were incubated with uptake buffer containing 0.4 μCi/mL [^3^H]-D-aspartate + unlabeled D-Asp 50 μM (Sigma–Aldrich, MO, USA). Uptake was stopped after 30 min of incubation by washing the cells with an ice-cold uptake buffer, and the incorporated radioactivity was evaluated as was mentioned previously. Four independent experiments (*n* = 4) were performed in quadruplicates from four different passages.

#### Effect of SiO_2_-NPs on Kinetic Parameters

The kinetics parameters were evaluated after treating the cells with 4.8 μg/mL of SiO_2_-NPs for 30 min. Then, the medium was replaced with uptake buffer containing 0.4 μCi/mL [^3^H]-D-aspartate + different unlabeled D-Asp concentrations 0,10, 25, 50, 100, and 200 μM (Sigma–Aldrich, MO, USA). Finally, uptake was stopped after 30 min of incubation by washing the cells with an ice-cold uptake buffer, and the samples were processed as described above.

A robust nonlinear regression was used to fit a model to the experimental data and estimate the parameters of the Michaelis–Menten equation (GraphPad Prism Software, La Jolla California, USA). Three independent experiments (*n* = 3) were performed in quadruplicates (4 wells by condition) from three different passages.

### Statistical Analysis

Results are expressed as the mean ± SEM from a least three independent cultures. A one-way or two-way analysis of variance was carried out to determine significant differences between conditions followed by Dunnett’s multiple comparison or Bonferroni test, according to the results. For statistical analysis of kinetic experiments, *t*-tests were used. The probability of 0.05 or less was considered statistically significant. All the plots and analyses were performed with GraphPad Prism Software (La Jolla California, USA).

## Results

### Cytotoxic Effects of Silica Nanoparticles on Endothelial and Astrocyte Cell Line

**Fig. 1:**
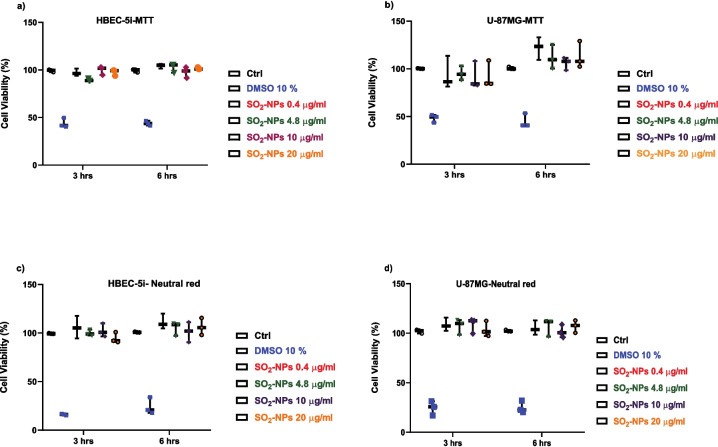
Cytotoxic effect of silica nanoparticles in HBEC (**a**, **c**) and U-87MG (**b**, **d**) cell lines. Both cell lines were treated with vehicle (control), DMSO 10%, or increasing SiO_2_-NP concentrations (0.4, 4.8, 10, and 20 µg/ml) for 3 and 6 h. **a**, **c** MTT assay. **b**, **d** Neutral red assay. DMSO at 10% was used as a positive control. Results are expressed as median and 25th and 75th interquartile percentiles of at least three independent experiments (*n* = 3), each tested in quadruplicate (two-way ANOVA followed by Bonferroni’s multiple comparison test)

In order to establish if SiO_2_-NPs trigger cytotoxic effects on HBEC or U-87MG cell lines, confluent cultures were exposed to different concentrations (0.4, 4.8, 10, and 20 µg/ml) of nanoparticles for 3 and 6 h, and cell viability was determined using MTT assay, based on the mitochondrial capacity to metabolize a formazan salt and the neutral red assay, which is based on the ability of viable cells to incorporate and bind the dye neutral red in the lysosomes. In HBEC, SiO_2_-NPs showed no cytotoxic effects across all the concentrations after 3 or 6 h of exposure (Fig. 1 a (MTT assay) and c (neutral red assay). In U-87MG cells, nanoparticles do not decrease the cell viability at any concentration used or time. The effect was the same in the MTT assay (Fig. 1b) and neutral red assay (Fig. 1d). These results allowed us to assess the effect of SiO_2_-NPs at the molecular level on the activity of glutamate transporters after SiO_2_-NPs exposure. Glutamate extracellular levels are regulated by a family of Na^+^-dependent glutamate transporters and excitatory amino acid transporters (EAATs) (Danbolt 2001). Five subtypes of transporters have been described and named EAATs 1 to 5. However, in HBEC-5i and U-87MG cell lines, the glutamate transporter system in terms of activity is not clear.

### Glutamate Transport Characterization

**Fig. 2:**
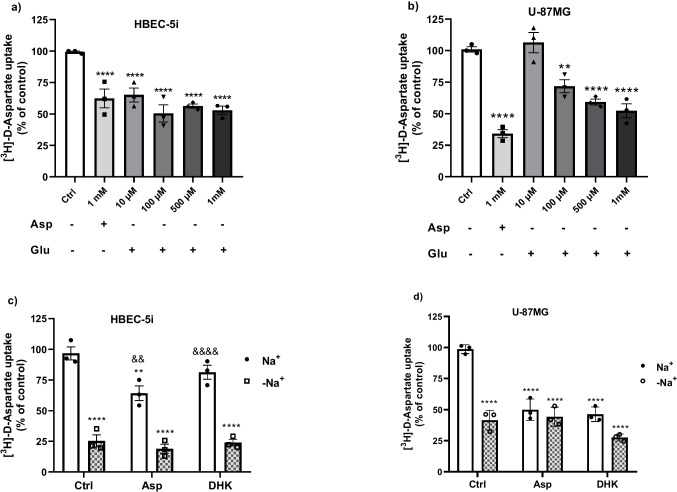
Characterization of glutamate transporters activity. Both cell lines were treated with aspartic acid (1 mM) and glutamic acid (10, 100, 500 µM and 1 mM) for 30 min (**a**, **b)**. Then, the medium was replaced with a buffer containing [^3^H]-D-Asp fand; its uptake was evaluated for 30 min in HBEC (**a**, **c**) and U-87MG (**b**, **d**) cells. Results are the mean ± SEM of at least three independent experiments (*n* = 3); each experiment was performed in quadruplicates. *p < 0.1, **p < 0.01, ***p < 0.001, ****p < 0.0001 vs control. In each plot, the Ctrl group was used as a standard condition; each bar was compared vs the control group. One-way ANOVA followed by Dunnett’s multiple comparison test was used in **a** and **b**. Two-way ANOVA followed by Tukey multiple comparison test was used in **c** and **d**. ****p < 0.0001 vs control with sodium, &&*p* < 0.001, &&&&*p* < 0.0001 vs control group without sodium

In endothelial cells from bovine and porcine brains, the expression of glutamate transporters (EAAT1, 2, and 3) has been reported (Helms et al. 2012; Cohen-Kashi-Malina et al. 2012), while in astrocytes, EAAT1 and 2, are known to be expressed (Rodríguez-Campuzano and Ortega 2021). We asked ourselves if these proteins are present in both cell lines and respond to aspartate or glutamate which have been reported to regulate the EAAT uptake activity. In both cell lines, the uptake is downregulated by aspartate and glutamate (Fig. 2a and b), an effect that has been reported by our group. Taking into consideration that EAAT2 has been reported in both cells (endothelial and astrocytes) and this is Na^+^-dependent, we used a selective blocker of EAAT2 dihydrokainic acid (DHK 100 µM) to evaluate [^3^H]-D-aspartate uptake activity. DHK did not decrease the [^3^H]-D-aspartate uptake, suggesting that EAAT2 does not mediate glutamate uptake in HBEC cells (Fig. 2c). On the other hand, on the astrocytic cell line, DHK reduces by approximately 50% the activity, which indicates EAAT2 is participating in the uptake process (Fig. 2d). Note that the absence of NaCl (-Na^+^) reduces the uptake up to 75% in HBEC cells and 50% in astrocyte cells, demonstrating most of the glutamate transport is Na^+^-dependent and carried out, probably by EAAT1 or 3 in HBEC cells and EAAT1 and 2 in astrocytes (Table 1).

**Table 1:** EAATs, localization, substrates, and inhibitors

Gene	Protein	Type of cell	Cellular localization	Substrates	Inhibitors	References
SLC1A1	EAAT3 (EAAC1)	Neurons Brain endothelial cells	Plasma membrane	D-Aspartate L-glutamate	TBOA (4S/5S)-POAD	Kanai and Hediger (1992) Helms et al. (2012)
SLC1A2	EAAT2 (GLT-1)	Astrocytes Glia cells Brian endothelial cells	Plasma membrane	D-Aspartate L-glutamate	TBOA Dihydrokainic acid WAY-855	Pines et al. (1992) Helms et al. (2012)
SLC1A3	EAAT1 (GLAST)	Glia cell Astrocytes Brain endothelial cells	Plasma membrane	D-Aspartate L-glutamate L-cysteine	TBOA UCPH-101	Storck et al. (1992) Helms et al. (2012)
SLC1A6	EAAT4	Neurons (Purkinje cells	Plasma membrane	L-glutamate	TBOA	Lin et al. (1998)
SLC1A7	EAAT5	Retina	Plasma membrane	L-glutamate	TBOA THA	Arriza et al. (1997) Shimamoto (2008) Vandenberg and Ryan (2013)

**Fig. 3:**
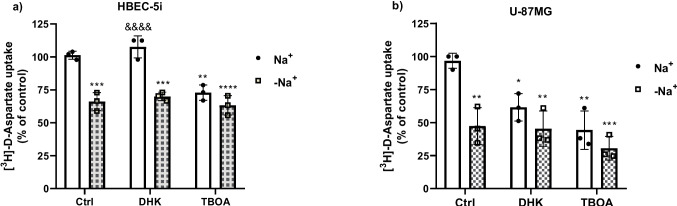
Pharmacological characterization of the glutamate transporter activity in HBEC and U-87MG cell lines. The cells were pre-treated for 30 min with EAAT2 selective inhibitor (DHK 100 µM) and unspecific EAAT1, 2, and 3 (TBOA 100 µM), and then, the medium was replaced with a buffer containing [^3^H]-D-Asp fand; its uptake was evaluated for 30 min in the presence or absence of sodium. Results are the mean ± SEM of at least three independent experiments (*n* = 3). *p < 0.002, **p < 0.001, ***p < 0.0002, ****p < 0.0001 compared vs the untreated control group. &&&&*p* < 0.0001 vs control group without sodium. Two-way ANOVA followed by the Tukey multiple comparison test was used

Endothelial and astrocyte cells have been reported to express EAAT1. So, next, we compare the effect of EAAT2 blocker, DHK, and TBOA, a non-specific potent inhibitor of EAAT1, 2, and 3, in the presence and absence of Na^+^. As depicted in panel a of Fig. 3, it was confirmed that DHK had no effect on the uptake, but TBOA decreased the uptake by around 30%, which suggests that in HBEC cells, the main transporter is EAAT1. In U-87MG cells, DHK decreased by 50% of the uptake as we observed previously. The TBOA-treated group showed a higher decrease, 35 ± 5%, as a sign of EAAT1 activity (Fig. 3b). These results suggest that in endothelial cells, the main glutamate transport is EAAT1, as has been reported. While in astrocytes, EAAT1 and 2 are participating actively in the glutamate uptake.

### Kinetic Parameters of the Glutamate Transport

**Fig. 4:**
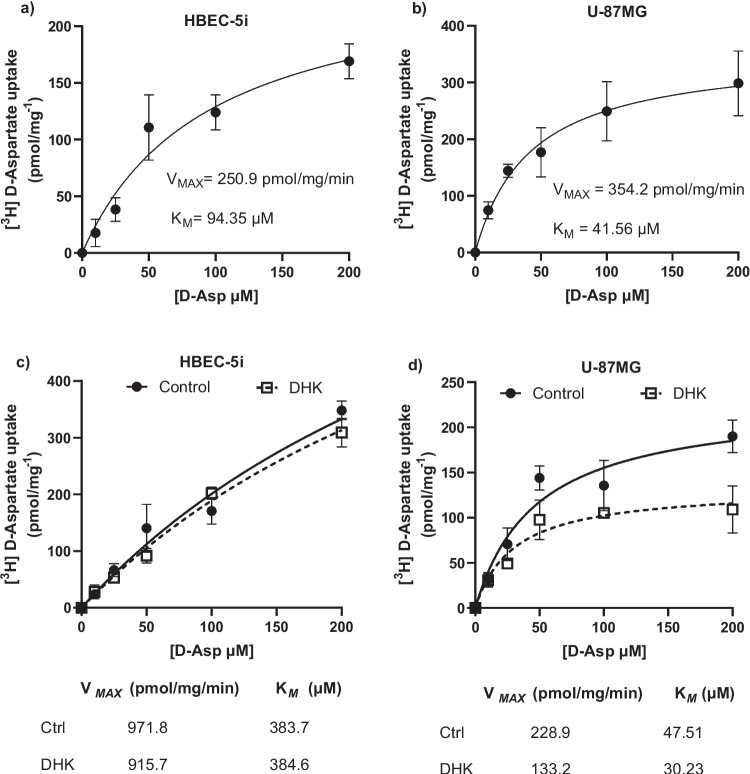
[^3^H]-D-Asp transport is saturable in HBEC and U-87MG cell lines. **a** HBEC. **b** U-87MG (b) cells. The monolayers were exposed to varying concentrations of D-Asp (ranging from 0 to 200 µM). Control or DHK-treated cells were used (**c**, **d**). Data are presented as the mean ± SEM from three independent experiments (*n* = 3) from three different passages, performed in quadruplicate. A robust nonlinear regression was used to fit a model to our data and estimate the kinetic parameters

We next characterized the kinetic parameters of [^3^H]-D-aspartate uptake in both cell lines. The concentration dependence of aspartate was evaluated, using cold aspartate ranging from 25 to 200 µM (Fig. 4a and b). Nonlinear regression analysis was used to determine Km and Vmax values. The results showed Km and Vmax values of 94.3 µM and 250.9 pmol/mg prot/min and 41.5 µM and 354 pmol/mg prot/min in HBEC and U-87MG cells, respectively. To further characterize the [^3^H]-D-aspartate uptake and determine whether the EAATs are mediating [^3^H]-D-aspartate uptake, we used the EAAT2 non-transported inhibitor, DHK. We found that 100 µM of DHK did not modify the kinetics parameters in HBEC cells, while in the U-87MG cell line, DHK modified Km values from 47.5 to 30.2 µM and reduced Vmax values from 228.9 to 133.2 pmol/mg prot/min. Suggesting, once again that, EAAT2 is the only active transporter in the U-87MG cell line.

### Silica Nanoparticle Exposure Decreases [^3^H]-D-Aspartate Uptake

**Fig. 5:**
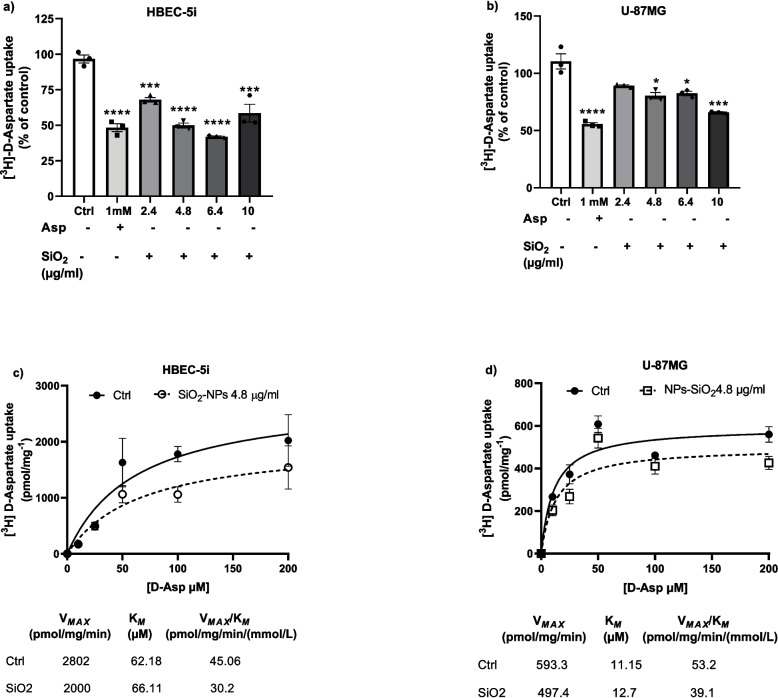
SiO_2_-NPs exposure decreases glutamate transport activity. Total [.^3^H]-D-Asp uptake was measured in control (Ctrl) or NPs-SiO_2_ treated cells, HBEC (**a**, **c**) and U-87MG cells (**b**, **d**). NPs-SiO_2_ concentrations used were as follows: 2.4, 4.8, 6.4, and 10 µg/ml for 30 min (**a**, **b**). Statistically significant differences between the control and experimental group are indicated by *p < 0.01, ***p < 0.001, ****p < 0.0001 versus control. In **c** and **d**, the cells were pre-treated with NPs-SiO_2_ (4.8 µg/ml) for 30 min and then treated with different D-Asp concentrations (ranging from 0 to 200 µM). A robust nonlinear regression was used to fit a model to our data and estimate the kinetic parameters. Data represent the mean ± SEM from three independent sets of cultures, performed in quadruplicate (one-way ANOVA followed by Dunnett´s multiple comparison test)

The results presented in Fig. 1 showed that exposure to SiO_2_-NPs do not reduce the viability at any of the concentrations used (0. 4, 4.8, 10, and 20 µg/ml) after 3 or 6 h. Therefore, we used this range of concentrations to evaluate whether SiO_2_-NPs disrupt the activity of excitatory amino acid plasma membrane transporters. Recent studies have shown that SiO_2_-NPs can cross the BBB (Liu et al. 2017; Liu et al. 2014), so a direct interaction with the cell membrane transport proteins might be taking place (Gilardino et al. 2015). To evaluate this possibility, the effect of the exposure to SiO_2_-NPs on both cell lines was undertaken. Confluent monolayers were incubated with different concentrations of particles (2.4, 4.8, 6.4, and 10 µg/ml) for 30 min. Then, [^3^H]-D-aspartate uptake activity was determined. The results show a decrease in the amount of [^3^H]-D-aspartate uptake into the cells after nanoparticle exposure. In HBEC cells, we observed a significant 40% decrease, with the four concentrations used (Fig. 5a), while in U-87MG cells, a consistent 30% reduction in the uptake was found at 2.4 and 4.8 µg/ml, and a 40% at 10 µg/ml (Fig. 5b).

To gain insight into the molecular mechanisms by which SiO_2_-NPs exposure decreases EAATs’ activity, we determined the kinetic parameters (Km and Vmax) of the aspartate transport in cells in control and SiO_2_-NPs-treated cells (4.8 µg/ml for 30 min). As depicted in panel c of Fig. 5, in HBEC cells, a decrease in Vmax and Km values from 2802 to 2000 (pmol/mg.min) and 62.1 to 66.11 (µM), respectively, was detected. When U-87MG cells were exposed to 4.8 µg/ml of SiO_2_-NPs, we observed a decrease in both kinetic parameters (Vmax and Km) from 593.3 to 497.4 (pmol/mg.min) and 11.15 to 12.7 (µM), respectively (Fig. 5d). One of the key events that can be determined with the kinetic parameters of the transport is uptake efficiency or catalytic efficiency (Vmax/Km), meaning the probability that an aspartate (glutamate) molecule bound to the transporter would be imported into the cytosol rather than be released back into the extracellular space (Rodríguez-Campuzano and Ortega 2021). As depicted in Fig. 5 panels c and d, a considerable decrease in the catalytic efficiency of the transport in SiO_2_-NPs-treated cells from 45 to 30.2 on HBEC and 53.2 to 39.1 on U-87MG cells was found.

### Silica Nanoparticles Target the EAAT1 Transporter

**Fig. 6:**
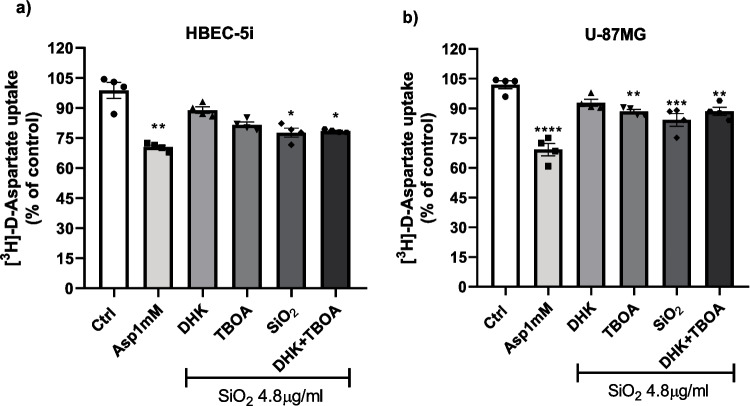
Glutamate transport EAAT1 is a target of silica nanoparticles. HBEC (**a**) and U-87MG (**b**) cells were pre-treated with the glutamate transporters blockers (DHK and TBOA), and [.^3^H]-D-Asp uptake was measured in control (Ctrl) and NPs-SiO_2_ treated cells (4.8 µg/ml) for 30 min. Statistically significant differences between the control and experimental group are indicated by *p < 0.1, **p < 0.01, ***p < 0.001, ****p < 0.0001 versus control. Data represent the mean ± SEM from three independent sets of cultures performed in quadruplicate (one-way ANOVA followed by Dunnett´s multiple comparison test)

As suggested from the results depicted in Fig. 3a and b, the main transport in both cell lines is EAAT1, since TBOA decreased the [^3^H]-D-aspartate uptake, but at least, in the U-87MG cell line, EAAT2 is present. We have observed that SiO_2_-NPs decrease the [^3^H]-D-aspartate (Fig. 5). Therefore, we asked ourselves if the nanoparticles could have a selective effect on a specific glial EAAT transporter, namely, EAAT1 or EAAT2. To this end, we used DHK, an EAAT2-specific blocker, and TBOA, a non-specific potent inhibitor of EAAT1, 2, and 3. In HBEC cells, there was a slight but non-significant decrease in the [^3^H]-D-aspartate uptake when TBOA and SiO_2_-NPs were present; however, with the co-treatment of DHK and TBOA, we observed a significant decrease around 20% (Fig. 6a). In sharp contrast, in U-87MG cells (Fig. 6b), there is a significant uptake decrease, approximately 20%, in the presence of TBOA and SiO_2_-NPs; the same effect was observed in the group treated with DHK plus TBOA. In both cell lines, the EAAT2 blocker did not have a significant effect. These results suggest that EAAT1 is the silica nanoparticle’s target.

## Discussion

SiO_2_-NPs have been proposed as an innocuous vehicle for drug targeting in the CNS. Nevertheless, SiO_2_-NPs are components of airborne particulate matter (PM) and exposure has been associated with a variety of health issues. For instance, cohort and in vitro studies have shown that exposure to fine, ultrafine PM in airborne pollution, and engineered nanoparticles may result in neurodegeneration and cognitive impairment (Heusinkveld et al. 2016). Several studies considered that amorphous silica is less toxic than crystalline silica. However, the effects of brain exposure to SiO_2_-NPs are not fully understood, most of all, those effects concerning to the potential damage of this material before being internalized. It has been shown that engineered nanoparticles reach the lungs and are deposited in several tissues, heart, liver, kidney, and CNS (Nel et al. 2006; Kleinman et al. 2008; Nemmar et al. 2004). Once nanoparticles arrive at the olfactory area, can cross BBB (Liu et al. 2017, 2014), be absorbed into the cerebrospinal fluid, enter the CNS, and cause possible damages (Oberdörster and Utell 2002), such as a direct interaction with the cell membrane that would not depend on SiO_2_-NPs internalization, being able to interact with transporter proteins (Gilardino et al. 2015). In the present contribution, we evaluated the effect of SiO_2_-NPs exposure on glial glutamate transporters’ activity expressed by BBB components, HBEC, and U-87MG cell lines (endothelial and astrocyte cells, respectively). The study of these nanoparticles could mimic the exposure to high levels of urban airborne PM and help us to understand the pollution effects in the human brain.

Controversial studies regarding nanotoxicity indicate that injection with SiO_2_-NPs (70 nm) induces liver injury, while 300- or 1000-nm nanoparticle size has no effect (Nishimori et al. 2009). These data indicate that the nanoparticle’s effects have not been fully defined, and most importantly, that studies of their safety are necessary. In the present contribution, we used SiO_2_-NPs, as a characterized model of PM, and HBEC and U-87MG cell lines, as BBB components, to investigate the potential toxicity of SiO_2_-NPs exposure, as a model system of atmospheric pollutants. The mechanism involved in SiO_2_-NPs toxicity is dependent on the size, dose, and cell type. Mesoporous silica can interact with cells in a size- and time-dependent manner (Orlando et al. 2017); particles with 100- to 500-nm size may disturb plasma membrane and result in cell rupture (Zhao et al. 2011).

The results from the MTT assays showed that SiO_2_-NPs at different concentrations (0.4, 4.8, 10, and 20 µg/ml) do not decrease the cell viability neither in HBEC nor U-87MG cells (Fig. 1a–d) after 3 and 6 h of exposure, allowing us to investigate potential adverse effects at the molecular level.

The precise glutamate levels in the brain’s extracellular fluid are kept in the micromolar range or avoid any potential excitotoxic effect that would disturb the proper physiological functioning of the CNS (Danbolt 2001). High glutamate concentrations are linked to neuronal death, and this process has been implicated with neurological deficits (Voss et al. 2021). Glutamate homeostasis studies in CNS have clearly demonstrated the critical role of astrocytes and the Na^+^-dependent high-affinity glutamate transporters. In this process, moreover, recent reports have suggested that endothelial cells participate in a coordinated brain-to-blood glutamate efflux and suggest that brain vasculature is also an integral participant in glutamate homeostasis (Cohen-Kashi-Malina et al. 2012; Gottlieb et al. 2003). In this context, we characterize here the glutamate uptake system in both cell lines. The present study demonstrates that HBEC cells express functional Na^+^-dependent EAAT1 (Fig. 2a and b), as previous studies have shown (Cohen-Kashi-Malina et al. 2012), while U-87MG cells express functional EAAT1 and 2 (Fig. 3a and b), as we observed by the DHK effect. Indeed, the transport is regulated by its own substrate (glutamate, Fig. 2c and d). The K_*M*_ for the Na^+^-dependent glutamate transport is in line with reported K_*M*_ values, Cohen–Kashi–Malina et al. (2012) found, in porcine endothelial cells, that in the presence of NaCl (total uptake), the K_*M*_ and V_Max_ values were 367 ± 15 µmol/L and 656 ± 21 pmol/mgprotein/minute, respectively. We observed in HBEC cells, a K_*M*_ of 94.3 µM and V_Max_ of 250.9 pmol/mg/min (Fig. 4a). In glial cells, mainly in astrocytes, K_*M*_ values range from 1 to 100 µmol, which depends on the transporter subtype and the assay system (Danbolt 2001; Vandenberg and Ryan 2013). We observed in U-87 cells a K_*M*_ of 41.5 µM and a V_Max_ of 354 pmol/mg/min, which is in line with the reported values.

After the characterization of the glutamate transport system in both cell lines, we explored the effect of the treatment of confluent HBEC and U-87MG monolayers with SiO_2_-NPs on their glutamate transporter activity. We observed a significant decrease in [^3^H]-D-aspartate uptake after the treatment of the cells with different SiO_2_-NP concentrations (Fig. 5a and b). To determine the kinetic parameters affected by this nanomaterial, we performed Michaelis–Menten saturation curves. We expected a decrease in V_Max_ and K_*M*_ which could explain the diminished in [^3^H]-D-aspartate uptake. We observed a decline in the kinetic parameters, triggered by SiO_2_-NPs, treated with 4.8 µg/ml (Fig. 5c and d). Surprisingly, the parameter that is known as *catalytic efficiency* (ratio between V_Max_ and K_*M*_) decreases upon the exposure to the nanoparticles. These results suggest that SiO_2_-NPs might interact directly with glutamate transporters present at the plasma membrane leading to a change in their uptake capacity. We therefore decided to gain insight into the possibility that the effect of nanoparticles would be specific for a particular type of glutamate transporter. To this end, we pre-treated the cells with EAAT2-specific inhibitor DHK or the broad-spectrum inhibitor TBOA (EAAT1, 2, and 3). A significant reduction in the uptake was observed in the groups treated with TBOA; although this reduction was more evident in U-87MG cells, these results suggest that EAAT1 or GLAST transporter could be the targets of SiO_2_-NPs (Fig. 6a and b).

**Fig. 7:**
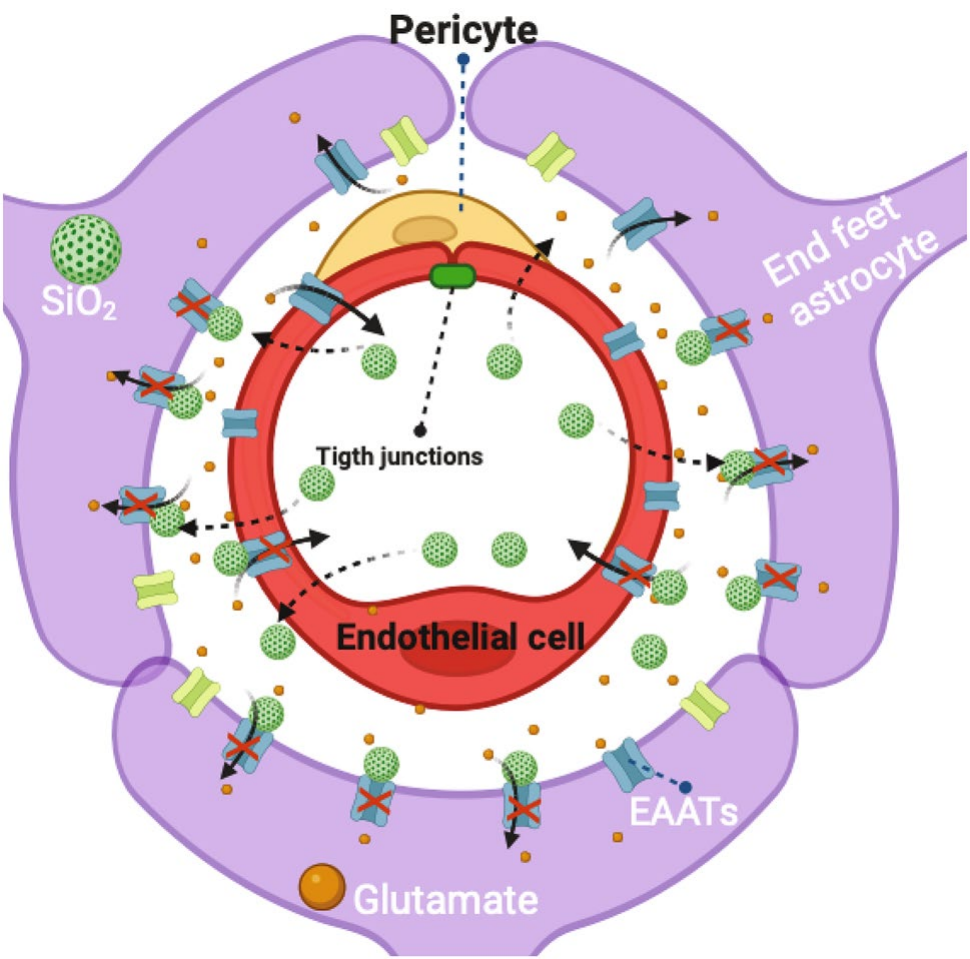
Summary of our current findings, depicting the effect of NPs-SiO_2_ exposure on glutamate transporters blocking their brain efflux capacity

Exposure to PM_2.5_ damages BBB in vitro triggering a neuronal cell death cascade through a macrophage-mediated toxicity and, also, induces the release of glutaminase which underlines the effects of neurotoxicity following PM_2.5_ exposure (Liu et al. 2015). Excitotoxicity is a phenomenon in which a disproportionate glutamate release takes place, over-stimulating its receptors and resulting in the activation of differential glutamate signaling pathways that lastly result in neuronal death (Voss et al. 2021; Skowrońska, et al. 2019). Once glutamate interacts with its receptors, it must be removed from the synaptic clef, and proteins in charge of the removal of glutamate are its plasma membrane transporters (Danbolt 2001). These transporters prevent the persistent activation of glutamate receptors, recycling the neurotransmitter and conferring protection from excitotoxicity. Zlotnik and co-workers demonstrated that BBB cell components participate in the glutamate efflux, from brain-to-blood, which increases after traumatic brain injury (TBI). Using blood glutamate scavengers, oxaloacetate and pyruvate, these authors showed neuroprotection after TBI (Zlotnik et al. 2012). Our results suggest that BBB components (endothelial and astrocyte cells) participate in the brain-to-blood glutamate efflux (Figs. 2, 3, and 4). Also, our results point out that exposure to environmentally relevant nanoparticle concentrations has toxicant effects, even before entering the CNS, at the BBB level; thus, the barrier is crucial to regulate solutes that maintain brain homeostasis. It is tempting to speculate that SiO_2_-NPs interact with the BBB and, once inside, decrease the glutamate uptake resulting in an increase in synaptic glutamate levels; the chronic accumulation of this neurotransmitter damages cognitive functions. This interpretation matches with epidemiological studies in populations from highly polluted cities, in which the experimental subjects show a clear deficit of cognitive functions (Calderón-Garcidueñas et al. 2002; Calderón-Garcidueñas and Ayala 2022); a summary of our findings is depicted in Fig. 7.

## Data Availability

No applicable.

## Abbreviations

BBB: Blood-brain barrier
BECs: Endothelial cells
CNS: Central nervous system
NVU: Neurovascular unit
TJs: Tight junctions
AD: Alzheimer disease
PD: Parkinson disease
JAMs: Junction adhesion molecules
ZO: Zonula occludins
TEER: Transendothelial electrical resistance
JACOP: Junction-associated coiled-coil protein
MAGI: Membrane associated guanylate kinase
AJs: Adherens junctions
PECAM: Platelet endothelial cell adhesion molecules
MRP: Multidrug-resistance associated protein
TGFβ: Transforming growth factor-β
GDNF: Glial-derived neurotrophic factor
bFGF: Basic fibroblast growth factor
AQP4: Water channel aquaporin 4
IL-1β: Interleukin-1β
TNF-α: Tumor necrosis factor-α
ABC: ATP-binding cassette transporters
GLUT1: Glucose transporter 1
MFSD2A: Major facilitator superfamily domain containing 2A
NKCC: Na^+^-K^+^-Cl^−^ cotransporter
Pgp: P-glycoprotein
Bcrp: Breast cancer resistance protein
LRP1: Lipoprotein receptor-related protein 1
SLCs: Solute carriers
PDGFR: Platelet-derived growth factor
VEGF: Vascular endothelial growth factor
Aβ: Amyloid beta
RAGE: Receptor for advanced glycation end-products
MMPs: Matrix metalloproteinases
HIF-1 α: Hypoxia-inducible factor-1α
DHA: Acid docosahexaenoic
HPA: Hypothalamic pituitary adrenal
S100β: S100 calcium binding protein B
METH: Methamphetamine
APC: Activated protein C
TBI: Traumatic brain injury
ALS: Ameotrophic lateral sclerosis
PAR1: Protease activated receptor 1
PI3K: Phosphatidylinositol-4,5-bisphosphate 3-kinase
HBMECs: Human brain endothelial cells
ISF: Insterstitial fluid
IL-6: Interleukin-6
NAc: Nucleus accumbens
HADC1: Histone deacetylase 1
APOE4: Apolipoprotein-E4
MS: Multiple sclerosis
